# Computational and transcriptional evidence for microRNAs in the honey bee genome

**DOI:** 10.1186/gb-2007-8-6-r97

**Published:** 2007-06-01

**Authors:** Daniel B Weaver, Juan M Anzola, Jay D Evans, Jeffrey G Reid, Justin T Reese, Kevin L Childs, Evgeny M Zdobnov, Manoj P Samanta, Jonathan Miller, Christine G Elsik

**Affiliations:** 1 Bee Power, LP, Lynn Grove Road, 16481 CR 319, Navasota, TX 77868 USA; 2 Department of Animal Science, Texas A&M University, College Station, Texas 77843, USA; 3 Bee Research Laboratory, USDA-ARS, BARC-E, Beltsville, MD, USA; 4 WM Keck Center for Interdisciplinary BioScience Training, Houston, TX 77005, USA; 5 European Molecular Biology Laboratory, Meyerhofstr., Heidelberg, Germany; 6 Systemix Institute, Los Altos, CA 94024, USA; 7 Department of Biochemistry, Baylor College of Medicine, Houston, TX 77030, USA; 8 The Institute for Genome Research, Rockville, MD 20850, USA; 9 Department of Genetic Medicine and Development, University of Geneva Medical School (CMU), rue Michel-Servet 1, 1211 Geneva 4, Switzerland

## Abstract

**Background:**

Non-coding microRNAs (miRNAs) are key regulators of gene expression in eukaryotes. Insect miRNAs help regulate the levels of proteins involved with development, metabolism, and other life history traits. The recently sequenced honey bee genome provides an opportunity to detect novel miRNAs in both this species and others, and to begin to infer the roles of miRNAs in honey bee development.

**Results:**

Three independent computational surveys of the assembled honey bee genome identified a total of 65 non-redundant candidate miRNAs, several of which appear to have previously unrecognized orthologs in the *Drosophila *genome. A subset of these candidate miRNAs were screened for expression by quantitative RT-PCR and/or genome tiling arrays and most predicted miRNAs were confirmed as being expressed in at least one honey bee tissue. Interestingly, the transcript abundance for several known and novel miRNAs displayed caste or age-related differences in honey bees. Genes in proximity to miRNAs in the bee genome are disproportionately associated with the Gene Ontology terms 'physiological process', 'nucleus' and 'response to stress'.

**Conclusion:**

Computational approaches successfully identified miRNAs in the honey bee and indicated previously unrecognized miRNAs in the well-studied *Drosophila melanogaster *genome despite the 280 million year distance between these insects. Differentially transcribed miRNAs are likely to be involved in regulating honey bee development, and arguably in the extreme developmental switch between sterile worker bees and highly fertile queens.

## Abstract 2

A total of 68 non-redundant candidate honey bee miRNAs were identified computationally; several of them appear to have previously unrecognized orthologs in the *Drosophila *genome. Several miRNAs showed caste- or age-related differences in transcript abundance and are likely to be involved in regulating honey bee development.

## Background

MicroRNAs (miRNAs) play pivotal roles in diverse biological processes through post-transcriptional regulation of gene expression. These short (approximately 22 nucleotide (nt)) non-coding RNAs repress protein synthesis by binding to partially complementary sites in the 3' untranslated regions (UTRs) of target genes [1-3]. MiRNAs affect biological phenomena such as cell proliferation, embryo and tissue differentiation [4], morphological change [5], and apoptosis, aging and life span [6]. Overall, miRNAs appear to regulate much of the coding transcriptome, influencing the spatial and temporal expression patterns of thousands of genes in plants, nematodes, insects, and vertebrates [7,8]. The pervasive influence of miRNAs exerts strong selective pressures on nucleotide sequences. Either positive selection for, or negative selection against, miRNA target sites can be detected in the 3' UTRs of most genes [9,10].

MiRNA sequences are often, but not invariably, highly conserved across great evolutionary distances, allowing identification of nearly identical short oligonucleotides that affect gene expression in species as divergent as worms and man [11]. This extraordinary sequence conservation may be indicative of extraordinary functional conservation, or some other exceptional evolutionary constraint. For instance, because a single miRNA may regulate hundreds of genes, mutation of a mature miRNA sequence could pleiotropically affect the expression breadth and specificity of many gene targets [12]. Thus, preservation of miRNA function in the wake of miRNA mutation would require coordinated compensatory mutation of each of its target's 3' UTRs - predicted to be an exceedingly rare confluence of events. Consequently, the sequence, structure and some functions of miRNAs may be conserved [13], while the specific gene targets and regulatory networks of particular miRNAs may exhibit significant interspecies variation [14].

The recently sequenced honey bee genome [15] provides an opportunity to detect novel miRNAs in this species and others, and to begin to infer the roles of miRNAs in key life history traits of honey bees, such as the development of fertile as well as sterile ('worker') individuals. Here we present the results of three independent computational surveys and transcriptional evidence for known and novel miRNAs. We suggest several novel miRNA candidates in honey bees. Some of these novel miRNAs appear to have been overlooked in analyses of the well-studied insect *Drosophila melanogaster *and other genomes.

## Results

### Computational identification of putative miRNAs

We exploited the whole genome assembly of the honey bee to predict candidate miRNAs. Three non-exclusive sets of miRNA candidates were compiled. First, honey bee sequences homologous to miRNAs listed in miRBase [16] were identified (HOM). Second, microconserved-sequence elements (MCEs), continuous sequences of lengths 22 through 29 nt that are common to and precisely conserved in all three of the *Apis mellifera*, *D. melanogaster *and *Anopheles gambiae *genomes, were catalogued [17].

Finally, slightly longer bee sequences (75-90 nt) sharing structural features characteristic of miRNAs and aligning well with similar sequences in *Drosophila *- an approach we call stem-loop scanning (SLS) - identified another set of putative honey bee miRNAs. This approach does not simply flag regions with propensity to form stem loop structures of appropriate length because there are thousands of such regions in the 235 Mb of the sequenced honey bee genome. Instead, Smith-Waterman alignments to regions of the *Drosophila *genome likely to form pre-miRNA structures were used to filter and refine the list of putative SLS candidates in honey bee.

Each putative miRNA precursor (pre-miRNA) identified by any method was folded to verify the thermodynamic propensity of the pre-miRNA sequence to adopt appropriate hairpin secondary structure - and to verify that the mature miRNA resided in the stem of the hairpin. We identified putative canonical honey bee miRNAs, but the MCE and SLS methods also suggested a number of possible new miRNAs, present but previously unrecognized in other genomes.

Consolidation of output from the MCE and homology-based miRNA search methods provided a final set of 65 unique miRNA candidate loci with 66 unique predicted miRNA models for experimental evaluation - including the best 25 predictions generated by MCE. This final set of 65 miRNA loci included 6 putative miRNAs identified by either homology or MCE methods, but also by the SLS process. However, none of the candidates identified only by SLS were among the final set of 65, or tested for expression in this study. Honey bee miRNA candidates, including some potentially novel miRNAs and a few honey bee orthologs of known miRNAs, are listed in Additional data file 1. There were two variant mature and precursor miRNA models predicted by MCE and HOM for one of the predicted miRNA loci. For each candidate honey bee miRNA model, Additional data file 1 gives the prediction method (HOM, MCE and/or SLS), miRBase designation if available, sequences of the putative mature honey bee miRNA and putative precursor region, genomic coordinates of each occurrence of mature and putative precursor miRNA sequences within the bee genome assembly release 4, location relative to coding sequence (CDS) of the honey bee official gene set [18] (intergenic, intronic, or overlapping a CDS), GC content of the GC content domain in which the miRNA is embedded (described in [15]), and folding energies. Folded precursors for some of the novel miRNAs are shown in Additional data file 8.

### Validation of honey bee miRNA candidates by RT-PCR

A variety of techniques are available for miRNA detection and validation, including hybridization techniques such as Northern blots and techniques using PCR (reviewed in [19]). We employed the RT-PCR technique described by Shi and Chang [20] to verify transcription of many of the candidate honey bee miRNAs we describe. In brief, this protocol invokes the polyadenylation of extracted RNA (in our case, after size-selection for small RNA species by either glass-fiber substrate binding or separation using polyacrylamide gel electrophoresis) followed by reverse-transcription primed by a poly(T) adapter. MiRNA-specific forward primers are then paired with a primer complementary to the RT adaptor for quantitative PCR amplification.

Table 1 shows normalized expression levels across a pool of larvae and adult bee samples for 30 candidate miRNAs. Some candidates were queried with multiple primers in order to test for strand-based expression and to distinguish between expression of precursor and mature miRNA sequences, leading to a total of 45 presented primers. Another 23 primers either generated artifactual PCR products in water or one-primer controls, or failed tests of amplification linearity. In general, candidates tested with forward and reverse primers showed much higher expression of one strand. As a methodological control showing strand specificity, primers for two variants of U4 spliceosome RNA (C5581a and C5581b) showed strong expression in the predicted reverse direction while a forward-oriented primer for C5581b showed almost no expression. Expression for this locus was marginal when the narrow (enriched for 18-30 nucleotide (nt) species) RNA pool was queried. Primers that matched mature miRNAs tended to generate stronger signal, especially when testing the gel-purified (18-30 nt) RNA extractions. Alignments of the tested primers to candidate miRNAs appear in Additional data file 2 and a gel showing quantitative RT-PCR (qRT-PCR) products from the 18-30 nt size selected RNA is found in Additional data file 9.

**Table 1:** Description of tested miRNAs

Locus	miRBase ID	Primer ID	Orientation	Location	Expression (not size selected)	Expression (size selected)
ame-mir-1	ame-mir-1	ame-mir-1.F	F	M	0.06	N/A
ame-mir-1	ame-mir-1	amir1.F	F	P	1.05	1.28
ame-mir-124	ame-mir-124	miR-124M351	F	M	2.09	0.69
ame-mir-124	ame-mir-124	miR-124M351R	R	M	0.40	0.07
ame-mir-2-1	ame-mir-2-1	ame-mir-2+.F	F	M	7.81	13.55
ame-mir-2-2	ame-mir-2-2					
ame-mir-2-3	ame-mir-2-3					
ame-mir-2-1	ame-mir-2-1	mir-2:1.1:101712.F	F	P	0.06	0.02
ame-mir-278	ame-mir-278	ame-mir-278.F	F	M	0.37	0
ame-mir-7	ame-mir-7	ame-mir-7.F	F	M	17.95	3.16
ame-mir-7	ame-mir-7	miR-7M112R	F	M	0.06	N/A
ame-mir-9a	ame-mir-9a	ame-mir-9a.F	F	M	23.69	9.58
ame-mir-9b	ame-mir-9b	ame-mir-9b	F	M	0.64	1.95
ame-mir-iab-4	ame-mir-iab-4	ame-miriab4.F	F	P	0.24	0.06
ame-mir-10	ame-mir-10	ame-miR-10	F	M	0.09	N/A
ame-mir-279	ame-mir-279	miR-279M341	F	M	5.53	1.04
ame-mir-279	ame-mir-279	miR-279M341R	R	M	0.56	N/A
ame-mir-283	ame-mir-283	HCmir-283.F	F	M	23.69	17.88
ame-mir-71	ame-mir-71	miR-71.R	R	M	1.70	1.38
ame-mir-87-2	ame-mir-87-2	mir-87:13.12:403730.F	F	P	0.43	0.91
ame-bantam	ame-bantam	banM365	F	M	0.16	0.06
C1504	ame-mir-925	C1504.F	F	M	0.11	0.30
C2989	ame-mir-926	C2989.F	F	M	31.25	N/A
C3345	ame-mir-927	contig3345.R	R	M	0.17	3.63
C4222	ame-mir-928	C4222.F	F	M	N/A	3.39
C5152a	ame-mir-190*	C5152a.F	F	O3	0.52	0.64
C5152a	ame-mir-190*	D+	R	O	1.29	0.20
C5152b	ame-mir-190	C5152b.F	F	P	0	0
C5152b	ame-mir-190	F-	R	P	76.96	25.28
C5303	ame-mir-137	C5303.F	F	O3	2.96	0.74
C5303	ame-mir-137	C+	R	O3	0.15	0.02
C5560	ame-mir-929	C5560.F	F	O	955,568	45,073
C5560	ame-mir-929	A-	R	O3	0.01	0
C5560	ame-mir-929	C5560.R	R	M	0.00	0
C5599	ame-mir-930	C5599b.F	F	M	0.54	0.08
C689	ame-mir-932	amir1.R	F	M	0.23	0.28
C689	ame-mir-932	contig689.F	F	P	20.62	N/A
C689	ame-mir-932	C689.F	R	M	0.16	N/A
C689	ame-mir-932	E+	R	M	N/A	1.69
C2187^†^		C2187.F	F	M	0.98	N/A
C2370^†^		C2370.F	F	M	8.37	N/A
C5581a^‡^		C5581a.R	R	M	20.62	N/A
C5581b^‡^		C5581b.F	F	M	0.07	0
C5581b^‡^		C5581b.R	R	M	22.10	N/A

Orientation is on predicted miRNA (F, forward; R, reverse). Location is within: mature miRNA (M); precursor sequences (P); overlapping mature miRNA with 3' primer end within mature sequence (O); overlapping mature miRNA but with 3' primer end in precursor (O3). Expression levels for pooled queen and worker samples are described in the text. The last two columns are normalized expression estimates for pooled RNA that either had or had not been size-selected by PAGE to include sizes from 18-30 nt. *C5152a is the reverse complement of ame-mir-190. ^†^C2187 and C2370 met thermodynamic criteria, but did not meet miRBase folding criteria. ^‡^Denotes U4 spliceosome RNA. The expression levels are scaled to the average of all primers.

We found 25 potentially novel miRNAs by MCE, of which 17 were tested by qRT-PCR. Twelve of these were expressed in one or more tissues, stages, castes or pooled RNA samples, while four had no detectable expression (C2327, C4131, C5267 and C6617). Nevertheless, three of the RT-PCR negative candidates showed evidence of transcription in the tiling array data (C4131, C6617 and C5267).

C5152a and C5152b are discrete miRNA predictions in physical proximity on opposite strands (shown in Additional data file 1) and both yield good hairpin predictions (Additional data file 8). C5152b is similar, but not identical, to *Drosophila *dre-ame-190. **:**Expression of C5152a and C5152b by RT-PCR was tested using multiple primers, and both F- and D+ primers showed expression (Additional data file 3). Primers F- and D+ were designed to amplify the mature miRNAs predicted for C5152a and C5152b, respectively (Additional data file 2). However, the complex overlap and antisense orientation of these two predictions, and binding sites for both F- and D+ within each of C5152a and C5152b, prevent us from excluding the possibility that only one is actually expressed in both sense and antisense orientations.

Overall, we provide evidence of transcription for most of the novel MCE predictions, including roughly two-thirds of novel candidates amenable to RT-PCR testing. Predicted expression levels were correlated between assays involving RNA extracts biased toward small species using either selective precipitation or electrophoretic separation (Table 1; Additional data file 3). Additional candidates will likely be confirmed as having transcription using other techniques and honey bee tissues or life stages.

### Validation of miRNA candidates by whole genome tiling array

We also analyzed the results of two whole-genome honey bee tiling array experiments for evidence that our candidate miRNAs were expressed. Using RNA pooled from multiple tissues and stages, genome-wide transcription, including intergenic regions, was evaluated by hybridization to 36-mer probes. Two strand-specific 36 nt oligonucleotide probes for every 46 bp of the honey bee genome were arrayed. The whole genome tiling array was hybridized in two separate experiments with two different pooled polyadenylated RNA samples; but the second experiment contained pooled RNA enriched for brain and thorax.

For each candidate miRNA, tiling probes in a genomic region containing its precursor sequence flanked by 50 bases on both 5' and 3' ends were examined. A miRNA was considered expressed if at least one probe within the chosen region measured signal above 90% of all tiling probes from the entire genome. Twenty-six miRNAs, listed in Additional data file 6, measured strong signal in either of the tiling array experiments and six in both. Among the latter six, C4222, C6617 and ame-mir-100 exhibited differential signal strength in the two tiling array experiments.

Tiling array experiments measure genome-wide expression patterns in an unbiased manner. In several organisms, signals from tiling arrays were observed in numerous noncoding regions of the genome, suggesting the presence of noncoding RNA, including tRNAs. Notably, tRNAs are approximately the same size as miRNA precursors [21]. However, neither pre-miRNAs nor mature miRNAs will be polyadenlylated. Thus, use of polyA RNA in these experiments therefore biased the RNA samples against mature miRNAs. Consequently, failure of some RT-PCR validated miRNAs to be detected as tiling array signals is not surprising. Conversely, there was difficulty in assigning statistical significance to the observed tiling array signals because the array experiments were designed to detect longer protein-coding genes. Therefore, there were too few probes (approximately 3-4) for each miRNA precursor, and typically only one of these probes showed strong signals. The significance of tiling array results is higher for the six miRNAs displaying strong signals in both experiments A and B, and for the twelve miRNA candidates that also exhibited RT-PCR results consistent with transcription. However, differential signal for three of the tiling array positive miRNA candidates suggests that those miRNAs (C6617, C4222 and ame-mir-100) may have roles in bee brain or thorax.

### Caste-, tissue- and age-related miRNA expression correlations

**Figure 1:**
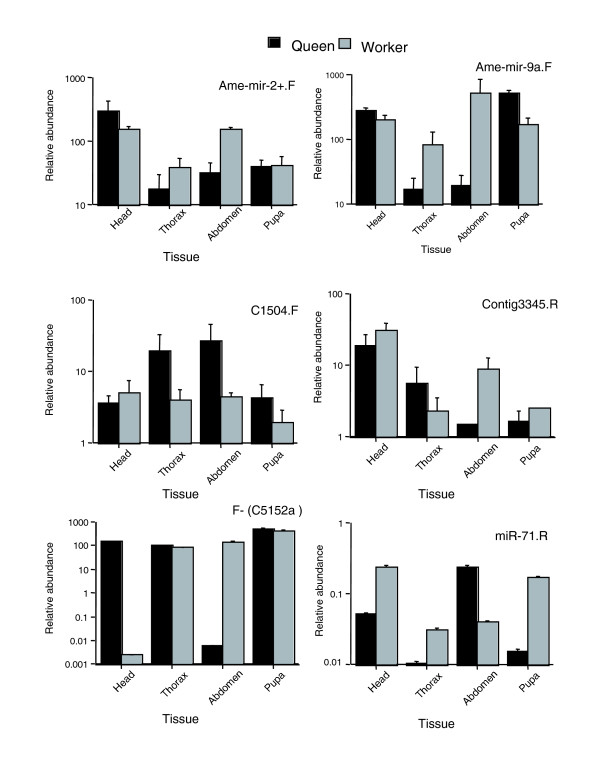
Normalized expression across worker and queen samples for six miRNA candidates. Values indicate relative expressions levels as log_10 _scale, with SD for three sample replicates, as described in the text. Primer IDs are indicated.

We hypothesized that miRNAs might be involved in the dramatic developmental fate changes associated with the switch from a reproductive female to a sterile worker female caste. Accordingly, RNA was isolated from various tissues and stages of both queen and worker honey bees and characterized by RT-PCR. Figure 1 and Additional data file 10 contrast expression levels for a subset of the candidate miRNA loci in adult head, thorax, abdomen and whole pupae, for both queens and workers. Several candidates showed differential expression between queens and workers in the abdomen, arguably the body part that is physiologically most distinct between these castes due to their different fecundity. Candidate loci ame-mir-9a, C3345, and C5152 were more strongly expressed in worker abdomens, while C1504 and ame-mir-71 were more strongly expressed in queen abdomens. Ame-mir-71 also had far stronger expression in developing (pupal) workers than in queens and in worker thoraces. A more complete summary of RT-PCR experiments for this subset is shown in Additional data file 4. In agreement with our hypothesis that computationally predicted honey bee miRNAs could be implicated in bee development, and particularly in the changes that characterize alternative fates of worker and queen, many miRNAs display tissue, stage or caste-related expression patterns. Additional data files provide the values of RT-PCR transcription estimates for pooled RNA (Additional data file 3) and additional queen/worker samples (Additional data file 4), primer sequences employed for experimental evaluation (Additional data file 5), and alignments of the primers to the precursor sequences (Additional data file 2).

### Intronic miRNAs and host genes

MiRNAs are often clustered within the genomes of mammals and flies, and this clustering is often associated with co-transcription of miRNAs and genes with which they are in close proximity [22]. The co-transcription of miRNAs and nearby genes may also reflect coordinate regulation of miRNAs and nearby genes. In particular, intronic miRNAs are often, though not invariably, coordinately expressed with their host gene and transcribed as a single primary transcript [23]. In support of the postulated role of miRNAs in regulating the alternative developmental trajectories associated with caste differentiation, we examined the functional role of honey bee official gene set genes in which intronic honey bee miRNAs are embedded [18]. Given the paucity of direct functional evidence for most genes in honey bees, we relied upon a comprehensive set of computational orthologs described elsewhere [15]. We discovered several notable relationships that will merit additional investigation. First, there were associations with fundamental cellular machinery of growth and development. Ame-mir-34, ame-mir-277 and ame-mir-317 all occupy intron 3 of GB10191. GB10191 is the ortholog of Rbp8 in *Drosophila*, and RPB8 in humans - part of the RNA polymerase II core complex and intimately involved in all transcriptional activity. Similarly, ame-mir-279 is embedded within intron 3 of GB12486, the honey bee DNA polymerase-α primase.

Intriguingly, the functional processes of other genes hosting intronic miRNAs suggest some bee miRNAs may be implicated in important but more complex caste differences. For instance, novel candidate miRNA C689 is found within GB10066, the bee ortholog of neuroligin, implicated in nervous system development. Novel miRNA C1504 is embedded in GB11212, whose *Drosophila *ortholog is involved in the dorsal/ventral patterning, expressed in wing discs, and negatively regulated by Ultrabithorax. Candidate C5267 is contained in GB15446, whose *Drosophila *homologs are regulators of transcription from RNA polymerase II promoters, and involved in eye development and other morphogenic interactions. Novel candidate C5599 is found within GB14516, the ortholog of *Dll *(Distalless), which has transcription factor activity and is intimately involved in proximal/distal pattern formation and morphogenesis, especially antennae and genitalia formation. Bee miRNAs may also be involved in programming behavioral response repertoires, as GB15597 harbors miRNA C4222, and its fly ortholog is *eag*, implicated in behavioral responses, including sensory perception of smell and flight.

### Gene Ontology analysis

We reasoned that an analysis of overrepresented Gene Ontology (GO) [24] terms associated with genes near miRNAs might offer additional insights into function for some bee miRNAs, and allow us to examine broad patterns of functional conservation between bee miRNAs and *Drosophila *miRNAs. We first determined the GO slim terms (a more general subset of GO terms) associated with the *Drosophila *ortholog of each bee gene [15]. Then using GeneMerge [25], we determined GO slim terms that were overrepresented among the set of bee genes occurring <10, <20, <50 or <100 kb from a predicted mRNA, compared with the set of all bee genes with *Drosophila *orthologs. Because some bee genes have multiple orthologs to *Drosophila*, and to ensure that our GO enrichment analysis was not biased by random selection of one to many fly orthologs of bee gene near miRNAs, we performed ten GeneMerge replicate experiments at each distance and report only GO terms whose Bonferroni corrected E-socres were less than 0.05 in all ten replicates.

GO analysis revealed the following: 'Physiological process' as the only GO term overrepresented among genes <10 kb from bee miRNAs in every replicate experiment; 'Response to stess' overrepresented in every replicate experiment for genes <20 kb from bee miRNAs; no GO term overrepresented in every replicate <50 kb from bee miRNAs; 'Nucleus' overrepresented in every replicate <100 kb from bee miRNAs. Running GeneMerge on a negative control set consisting of randomly selected bee genes yielded no GO terms with significant Bonferroni corrected E-scores.

To compare GO terms associated with these miRNAs in bee and fly, we conducted a similar analysis of *Drosophila *genes near miRNAs. We obtained GO slim terms associated with *Drosophila *genes occurring <10, <20, <50 or <100 kb from *Drosophila *orthologs of these bee miRNAs, and ran GeneMerge to find overrepresented GO terms. As before, only GO terms whose Bonferroni corrected E-scores were less than 0.05 in all ten replicate experiments are reported. The GO experiment data are summarized in Additional data file 7. Interestingly, the GO term 'Physiological process', which was overrepresented among bee genes <10 kb from miRNAs was also overrepresented among *Drosophila *genes <20, <50 and <100 kb from miRNAs. As before, running GeneMerge on a negative control set consisting of randomly selected *Drosophila *genes yielded no GO terms with significant Bonferroni corrected E-scores.

Compared to bee, there were far more GO terms that were significantly enriched among genes near miRNAs in the *Drosophila *genome. For example, four GO slim terms ('Development', 'Morphogenesis', 'RNA binding' and 'Signal transduction') were overrepresented in all replicates at every distance in *Drosophila*, and there were 29 GO terms significantly enriched among genes <100 kb from fly miRNAs (Additional data file 7). In contrast, in the bee genome, there were no GO terms enriched at every distance, and only 1 GO term ('Nucleus') enriched among genes <100 kb from bee miRNAs. This disparity between bee and *Drosophila *is likely caused by the increased sensitivity in the *Drosophila *experiment compared to the bee experiment. The *Drosophila *experiment used *Drosophila *GO annotations directly, whereas the bee experiment relied on the existence and detection of *Drosophila *orthologs for each bee gene.

## Discussion

The honey bee genome [15] offers a rich resource for investigation of the genomic networks and emergent systems that characterize sociality and enable coherent operation of the complex web of interactions in the hive. However, the significant level of sequence divergence of honey bee from *Drosophila *and mosquito, and the absence of closely related genome sequences suitable for phylogenetic shadowing can impede genomic comparisons involving bees. We turned evolutionary distance to our advantage, reasoning that strongly conserved sequences in an appropriate length range (MCEs) might represent previously undiscovered miRNAs (the MCE algorithm) [17]. In addition, we exploited the secondary structure characteristics of most confirmed miRNAs, and the conservation of core microprocessor components in bee, like Drosha, to identify other candidates that would adopt pre-miRNA hairpin structures, and produce significant Smith-Watermann alignments between putative bee and *Drosophila *miRNAs (the SLS algorithm).

Among those novel miRNA predictions we tested, we observed only one false positive candidate identification by MCE. C5581 was predicted as a miRNA, but that sequence is homologous to a U4 splicing RNA. There was one case in which two methods predicted slightly different miRNAs at overlapping genomic coordinates. Mature ame-mir-137, identified by HOM, is completely identical over the 22 nt that it overlaps with the 27 nt of mature C5303, predicted by MCE. We observed two cases where different miRNA predictions occurred at overlapping genomic coordinates, but the opposite strand: C5152a/C5152b (primers F- and D+) and ame-mir-9b/ame-mir-79. In both cases, at least one of the opposing strand pair was identical or similar to a known mature miRNA. Predicted ame-miR-9b and ame-mir-79 are identical to known miRNAs. Predicted mature C5152b is similar, but not identical to *Drosophila *dme-mir-190; C5152b is longer than dme-mir-190, and differs at only three nucleotides internally. These may be examples of miRNA sense/antisense transcription.

The SLS output contained five predictions with significant similarity to the HOM output (ame-mir-13a, ame-mir-276, ame-mir-305, ame-mir-92 and ame-mir-9a) and only two predictions with significant similarity to the top 25 MCE candidates, both of which were variants of C5152. Of these SLS predictions, only ame-mir-9a and C5152 were tested for expression by RT-PCR, and both were validated. The tiling array evidence we accumulated also suggests that mir-305 is expressed. The SLS output included several novel pre-miRNA predictions that contained apparent repeat motifs and are unlikely to be true miRNAs. However, other SLS candidates may represent new miRNAs and future experiments will more systematically assess evidence of expression for some of them.

We detected transcription of mature miRNAs as well as some pre-miRNAs. Generally, putative mature miRNA transcript abundance exceeded the level of precursor transcripts. Primers for mature miRNAs also tended to show the strongest effects of transcript direction (for example, ame-mir-279; Table 1), and retained strand-specific expression levels when the 18-30 nt RNA pool was assayed. Nevertheless, tests at a number of candidate miRNAs indicated fairly similar (<5-fold difference) transcription levels for both RNA strands (for example, ame-mir-1). Due to the small sample sizes, we have highlighted only the more extreme expression differences, although, as has been shown in expression studies of protein-encoding transcripts in bees, even subtle differences in transcript abundance could play important roles in development. It is possible that actual mature miRNA for those candidates that did demonstrate expression may differ slightly from the mature miRNA we predicted. For example, a variant of the primer for candidate ame-mir-7 (ame-mir-7.F) indicated a very strong transcript level, while a primer with one more 3' nucleotide (T; miR-7M112R) gave no product. Thus, we showed that our RT-PCR technique was very sensitive to small primer sequence differences, as shown in plant miRNAs by Shi and Chang [20].

Likewise, the strongest expression product observed (C5560F) was primed by a forward primer that stopped one base short of the 5' end of the predicted mature miRNA (Additional data file 2). Because it is possible that the actual novel mature miRNA sequences may differ slightly from the sequence of the candidate mature miRNA primers we tested, we cannot unequivocally reject those candidate miRNAs for which we did not obtain reproducible expression patterns.

Honey bee genomic study is still young, but initial observations offer some clarity and focus for further investigation. First, with a few notable exceptions (for example, odorant receptor genes and genes involved with innate immunity), there are as yet few potential relationships between gross genomic features and the social organization of bees [15]. In fact, the emergence of social life and its manifestation in bees may rely mainly on fairly subtle genomic interactions that affect gene network organization, regulation and expression patterns. In support of this hypothesis, previous work suggests that the development of distinct reproductive castes (workers and queens) in honey bees reflects the differential regulation of well-established developmental genes, rather than that of a parallel set of caste-specific genes [26,27].

We submit that miRNAs and their combinatorial interactions with overlapping and independent target gene sets may offer a tractable means to aid the evolution of sociality, by stabilizing the alternative developmental programs that generate distinct castes from a uniform genetic groundplan. Thus, the evolution of distinct reproductive and sterile castes might proceed from the loss or acquisition of miRNA binding sites in the 3' UTRs of particular genes by drift or selection, coupled with divergent temporal or spatial expression of miRNAs between workers and queens. In fact, it has recently been suggested that miRNAs may be understood as contributing to canalization and genetic buffering of gene regulatory networks by interacting with transcription factors in coherent and incoherent feed-forward loops to stabilize phenotypic variability [28]. However, we need not posit that miRNAs act as direct switches for differential developmental pathways. The same canalizing effect could be achieved with miRNAs acting as global regulators of tissue identity and gene expression breadth and specificity. Indeed, the properties that make miRNAs attractive candidates as stabilizers of phenotypic variability would also allow miRNAs to modulate emergence of different phenotypes upon alternative spatial or temporal expression in different castes. Two candidates showed especially strong expression differences between identical tissues from bee queens and workers (Figure 1). Ame-mir-9a.F was expressed most strongly in worker versus queen thorax and abdomen. Candidate 5152a was overexpressed in queen versus worker head, then showed the opposite pattern in the abdomen.

We also present many unrecognized miRNAs in honey bee and show that some of them, as well as other canonical miRNAs, appear to be transcribed in a stage-, tissue- or caste-specific manner (Figure 1). In fact, the genomic location of many of the most strongly caste, stage or tissue biased miRNAs, coupled with known functional activities of some miRNAs in other species, orders and phyla, allow inferences regarding the roles these caste- or stage-biased miRNAs may play in honey bees. For instance, we find that ame-mir-9a is among the most strongly caste-biased miRNAs, with much higher expression levels in adult worker thorax and abdomen than similar queen tissues, but higher levels of mir-9a occur in queen pupae (Figure 1). Interestingly, mir-9a controls sensory organ precursors (SOPs) in *Drosophila*, with loss of mir-9a function resulting in ectopic production of SOPs, while overexpression of mir-9a yields a severe diminution of SOPs. Mir-9a is also expressed at high levels in epithelial cells adjacent to SOPs in proneural clusters, suppressing *sens *through miRNA/target interactions in the *sens *3' UTR, and inhibiting neuronal fate in non-SOP cells [29]. This suggests possible roles for ame-mir-9a in influencing caste differences in honey bees. Another example is C1504.F, which is expressed in higher levels in queens than workers (Figure 1) and is nested within the honey bee ortholog of the RNA binding protein gene, CG32062. Expression of CG32062 in *Drosophila *is dependent upon Notch-mediated signaling from the Dorso-Ventral organizer (D/V) boundary, and repressed by the homeotic gene, Ultrabithorax. The product of CG32062 likely constitutes a second long-range D/V morphogen, independent of Wingless (Wg) [30]. MiRNAs in other organisms are often organized in clusters that lie in physical proximity in the genome, and may be present in multiple copies too. In *D. melanogaster*, the proapoptotic K-box miRNA mir-2, and mir-13 occur jointly. The same relationship holds in bees, and ame-mir-71 is also present within this same region (Table 1). In fact, even with a relatively fragmented genome consisting of over 9,000 scaffolds, we can discern that the honey bee harbors several linked sets and/or multiple copies of miRNAs. They include ame-mir-1, which is near ame-mir-133. We note that mir-1 and mir-133 are co-located in physical proximity in organisms as diverse as honey bees, frogs, mice and men, and are well-documented regulators of myogenesis in other organisms [31]. Ame-mir-1 and ame-mir-133 may exhibit similar functions in honey bees. Other examples of clustered miRNAs or multicopy miRNAs include: novel miRNA C5152a antisense to C5152b; novel C5303 overlapping ame-mir-137; ame-mir-9b overlapping the ame-mir-79 locus, but on the opposite strand; ame-mir-12 near ame-mir-283; ame-mir-275 near ame-mir-305; ame-mir-277 near ame-mir-317 and ame-mir-34; C1504 near ame-mir-375; and ame-let-7 on the same scaffold as ame-mir-100. Two of the most interesting cases involve multiple miRNAs in the introns of single genes. Ame-mir-277, ame-mir-317 and ame-mir-34 occur in the same intron of GB10191 - a core component of the RNA polymerase II complex. Finally, three copies of ame-mir-2, plus one instance each of ame-mir-13a and ame-mir-71, all occur within intron 3 of GB15727 - a serine/threonine phosphatase lost from *Drosophila*, but with both vertebrate and more ancient metazoan orthologs.

That fact that we found three GO terms ('Physiological process', 'Nucleus' and 'Response to stress') that were overrepresented among genes near miRNAs in both the *Drosophila *and bee genome demonstrates that some miRNAs function in the same or similar functions in *Drosophila *and bee. Furthermore, this result allows us to ascribe roles for honey bee miRNAs in processes relevant to these GO terms. Future studies of the specific genes near these miRNAs and annotated with these GO terms may help elucidate how these miRNAs function in honey bee.

The sensitivity of the GO experiment in bee was limited by a number of factors. The GO analysis considers only those bee genes with recognizable orthologs in *Drosophila*, and the GO annotation for bee genes was always based upon functional evidence from *Drosophila*. Furthermore, in honey bee, the GeneMerge E-score for GO terms present in every experiment varies somewhat depending upon the particular *Drosophila *ortholog selected for use in GeneMerge, at least when there is more than one *Drsophila *ortholog. While 'Development', 'Morphogenesis', 'RNA binding' and 'Signal transduction' were overrepresented in every *Drosophila *experiment at all distances, there are no GO terms overrepresented in every bee experiment at each distance. Therefore, we suggest that the lack of enrichment for these same GO slim terms in the bee experiment may reflect the lack of a complete gene list in honey bee, the paucity of direct functional evidence for honey bee genes, and the reliance upon *Drosophila *orthology and GO annotation for bee genes. As honey bee genome annotation and functional genomics proceeds, further GO analysis may reveal additional functional attributes for honey bee miRNAs.

## Conclusion

Not surprisingly, the honey bee genome contains numerous candidate miRNAs that can be identified by computational methods. We show that some honey bee candidates identified in this way have been overlooked in other genomes. Some novel and canonical miRNA transcription levels differed strongly across the tested tissues and samples. Honey bees and other social insects are defined by a developmental polymorphism between highly fertile, long-lived queens and largely sterile workers. Differences in miRNA expression observed in homologous tissues of queen and worker may help provide insights into gene regulation during the remarkable developmental switch characterizing caste differences in the honey bee.

## Materials and methods

### Computational miRNA predictions

Our first strategy for identifying novel miRNAs invoked BLASTN searches of known miRNAs from miRBase release 8.0 [16] against the honey bee genome (Assembly release 4.0) using wordsize 7 and E-score threshold ≤0.1. These searches identified several hundred candidate bee miRNAs with significant matches to miRNAs from other species. A sliding window of 110 nt with increments of 3 nt was scanned along the sequences extracted at 100 nt upstream and downstream of each match. Windows were scored for folding energy (at least 25 Kcal/mol) using RNAFOLD [32], then for base pairing and position of putative mature miRNA along the stem. Candidates with at least 16 bases paired to the opposite strand were considered putative mature regions. Windows that passed this scoring scheme were visually inspected for proper folding.

Our second strategy relied on three-way, all against all, genomic comparisons of *D*. *melanogaster*, *A*. *gambiae and A. mellifera *to identify probable honey bee miRNA candidates [17]. Hundreds of microconserved MCE sequences identified in this way included more than 40% of previously validated *Drosophila *miRNAs, and this set seems likely to contain additional and novel miRNAs shared by bee and *Drosophila*. The secondary structural features of known pre-miRNAs in *Drosophila *are expected to be characteristic of novel pre-miRNAs of bee as well, because the genes involved in processing primary RNA transcripts into mature miRNAs in *Drosophila *are conserved in honey bee. Consequently, secondary structures of candidate bee miRNA precursors were screened for proper folding and thermodynamic stability typical of *Drosophila *miRNA precursors, and putative mature miRNAs were eliminated if they did not lie within the stem regions of the pre-miRNA hairpins, according to the criteria previously proposed by Ambros *et al*. [33]. Ground-state energies and structures were computed with the Vienna Package [34].

For the third strategy we applied a novel algorithm, SLS, to the entire honey bee genome to identify sequences that would adopt appropriate hairpin secondary structure. In the SLS method, overlapping 100 nt segments of the genome are analyzed for sequences that can form loops similar to those seen in known miRNAs. In detail, each 100 nt segment was aligned to its reverse complement using a modified Smith-Waterman alignment algorithm (G::T pairing was penalized less than other mismatches). Good alignments were tested to determine if they would form a stem and a loop with size typical of known miRNAs. Specifically, stems had to be 20-25 bp, and loops had to be 4-35 nt. Candidate sequences were then subjected to thermodynamic testing using Mfold [35] to determine free energy values. Those with folding energies less than -20 kcal/mole were discarded. This entire process was performed on both the honey bee and *Drosophila *genomes. Putative miRNAs from honey bee that aligned well to putative miRNAs from *Drosophila *were saved as candidate miRNAs.

### Transcriptional analyses: RT-PCR

RNA was extracted and enriched for short transcripts using a variant of the RNAqueous (Ambion, Austin, TX, USA) protocol. Honey bee tissues (head, thorax, and abdomen from queens and workers, and whole bodies from queen and worker prepupae) were ground in 200-600 μl lysis grinding buffer depending on tissue volume. This suspension was diluted in an equal volume of 64% EtOH and then spun through the provided filter columns. The flow-through, containing smaller RNA species, was then mixed with a 70% volume of isopropanol and passed through a second filter column in order to trap the now-precipitated small RNAs. After prescribed wash steps, RNA was eluted from this second column in 50 μl sterile H_2_O. RNA size range and quantity was estimated using an Agilent 9000 Bioanalyzer (Agilent Technologies, Santa Clara, CA, USA). A second extraction was carried out as above for queen and worker head, thorax, and abdomen, as well as third-instar larvae and prepupal bees. This extraction was separated using a 15% denaturing (TBE-urea) polyacrylamide gel (Invitrogen, Carlsbad, CA, USA). RNA species 18-30 nt in length were cut from the gel, eluted as a group using a FLASHPAGE mini-electrophoresis unit (Ambion), purified by EtOH precipitation, and resuspended in 50 μl sterile H_2_O.

Contaminating DNA was removed by exposing 2 μg of each total RNA pool to 10 U DNaseI with appropriate buffer (Ambion) in the presence of 20 U RNAsin (Roche, Mannheim, Germany). Samples were incubated 1 hour at 37°C, then 75°C for 15 minutes. Polyadenylated tails were added to all transcripts using a 15 μl reaction containing 2 μg total RNA, 2 U E-PAP enzyme with appropriate 1× buffer (Ambion), 4 mM MnCl_2_, and 1.7 mM ATP. Samples were incubated at 37°C for 1 hour. cDNA was prepared from 0.4 μg polyadenylated RNA template in a 15 μl reaction containing 10 pmol oligo-dT linker (5'GCG AGC ACA GAA TTA ATA CGA CTC ACT ATA GGT_12 _VN) and 2 mM dNTP. The reaction was heated to 70°C for 10 minutes and placed on ice. After pre-heating to 42°C for 2 minutes, 4 μl of reverse transcriptase mix, containing 50 U Superscript II in appropriate buffer and reagents (Invitrogen) was added. Synthesis was carried out at 42°C for 50 minutes, followed by 15 minutes at 70°C.

The above cDNA was diluted 1:5 and used as the template for amplification in an iCycler real-time PCR thermalcycler (Biorad, Hercules, CA, USA). Gene specific primers for approximately two thirds of the putative miRNAs were designed based on the predicted mature or precursor RNA sequences (Table 1). The 25 μl reaction mixes consisted of 1 U *Taq *DNA polymerase with appropriate buffer (Roche), 1 mM dNTP mix, 2 mM MgCl_2_, 1× SYBR Green dye (Molecular Probes, Eugene, Oregon, USA), 10 nM Fluorescein calibration dye (Biorad), and 0.2 μM of each forward and reverse primer. The thermal program for all reactions was 95°C for 30 s followed by 40 cycles of (95°C for 30 s, 60°C for 30 s, 72°C for 30 s, 76°C for 10 s immediately after the extension step for fluorescence capture). Melt-curve analysis and agarose gel analyses were used to test whether PCR products were the appropriate size (gel products 60-80 bp, dissociation temperatures 76-81°C). In addition, qPCR runs using negative (no template) templates, as well as miRNA forward primers without the adaptor primer, were used to exclude primers that showed signs of spurious amplification (*n *= 23).

Threshold cycle (C_T_) values for each miRNA were subtracted from the mean C_T _values for all miRNAs surveyed in a given cDNA. Amplification efficiency (serial dilution) analyses suggested that these PCR reactions were highly efficient and, accordingly, relative abundances were calculated as 2^δCT^. While the low replicate number precludes statistical analyses, means and standard deviations are presented for the two sample replicates in order to indicate sample variability.

### Gene ontologies of miRNA-regulated genes

To determine the functional categories of bee and fly genes under control of miRNAs, we looked for GO terms [24] overrepresented among genes in close proximity to putative miRNAs in the *Drosophila *and bee genomes. GO slim terms and annotations for *D. melanogaster *genes generated at FlyBase [36] were obtained from the Gene Ontology Consortium website [37]. GO terms were assigned to genes of the honey bee Official Gene Set [18] using *D. melanogaster *orthologs, which were identified as described by the Honey Bee Genome Sequencing Consortium 2006 [15]. In cases where more than one fly ortholog existed for a given bee gene, a random fly ortholog was selected independently in each replicate experiment. GeneMerge [25] was then run using test sets of genes <10 kb, <20 kb, <50 kb or <100 kb from putative miRNAs and their associated GO slim terms, and a population set consisting of all mapped bee genes with fly orthologs (for the bee experiment) or all mapped fly genes (for the fly experiment). Ten replicate experiments were conducted for both fly and bee analyses, and only GO terms whose Bonferroni corrected E-scores were less than 0.05 in all ten replicate experiments were considered significantly overrepresented. For negative control experiments, GeneMerge was run on a test set of randomly selected bee or fly genes equal in number to the set of bee or fly genes <10, <20, <50 and <100 kb from a putative miRNA.

## Additional data files

The following additional data are available with the online version of this article. Additional data file 1 provides a complete listing of candidate miRNAs, including miRBase designation, the sequence of the putative mature honey bee miRNA, the sequences 110 nt up- and downstream of each mature miRNA candidate (putative precursor region), the genomic coordinates of each occurrence of mature and putative precursor miRNA sequences within the bee genome assembly release 4, whether the candidate miRNA is intergenic, intronic, or overlapping a CDS, the GC content of the GC content domain in which the miRNA is embedded (described in [15]), and folding energy. Additional data file 2 provides alignments of primers to precursor miRNAs. Double strands of precursors are shown, with mature miRNA indicated as lower case embedded in the sense strand, which is otherwise uppercase. Note that reverse primers are shown in the 3' to 5' direction, to show alignment to the sense strand. Additional data file 3 provides qPCR expression results from gel-purified 18-30 nt RNA extractions from pooled tissues (head, thorax, and abdomen from queens and workers, and whole bodies from queen and worker prepupae). The ID column corresponds to gel lanes in part b in Additional data file 9. C_T _is a predetermined threshold at which fluorescence from PCR products exceeds background fluorescence. Additional data file 4 provides mean expression values for queen and worker honey bee tissue-specific samples. Additional data file 5 provides the primers employed in expression analyses, and expression estimates for pooled samples. Additional data file 6 provides miRNA tiling array probes with hybridization signal in the top 10% of tiling array results in two independent experiments. The RNA sample from array B had a higher concentration of bee brain and thorax than the first. Additional data file 7 provides GO analysis of genes located within 10, 20, 50 and 100 Kb of miRNAs. There is a different worksheet containing the results of 10 replicates each, for honey bee and *Drosophila*. Each worksheet provides E-score, Bonferroni Corrected E-score, and GO term. Additional data file 8 shows folded hairpins for precursors of novel honey bee miRNAs. Additional data file 9 provides two figures: Figure A shows a 15% PAGE separation of small-enriched RNAs from honey bee queen head, thorax, and abdomen, and worker head, thorax, and abdomen. RNA sized at 18-30 nt was excised from the gel and purified for qPCR as described in the text. The left lane shows a 10 nt RNA size marker, with the 10 nt band at bottom left. Figure B shows the size variation of PCR products generated from small-enriched RNA pools and candidate primers. Most products were approximately 75-90 bp in length. Alphanumeric label refers to sample ID as described in Additional data file 3. Additional data file 10 shows normalized expression across worker and queen samples for additional miRNA candidates. Values indicate relative expressions levels as log_10 _scale, with SD for three sample replicates, as described in the text.

## Supplementary Material

Additional data file 1 Includes miR designation, the sequence of the putative mature honey bee miRNA, the sequences 110 bp up- and downstream of each mature miRNA candidate (putative precursor region), the genomic coordinates of each occurrence of mature and putative precursor miRNA sequences within the bee genome assembly release 4, whether the candidate miRNA is intergenic, intronic, or overlapping a CDS, the GC content of the GC content domain in which the miRNA is embedded (described in [15]), and folding energy. Click here for file

Additional data file 2 Double strands of precursors are shown, with mature miRNA indicated as lower case embedded in the sense strand, which is otherwise uppercase. Note that reverse primers are shown in the 3' to 5' direction, to show alignment to the sense strand. Click here for file

Additional data file 3 The ID column corresponds to gel lanes in part b of Additional data file 9. C_T _is a predetermined threshold at which fluorescence from PCR products exceeds background fluorescence. Click here for file

Additional data file 4 Mean expression values for queen and worker honey bee tissue-specific samples. Click here for file

Additional data file 5 Primers employed in expression analyses, and expression estimates for pooled samples. Click here for file

Additional data file 6 The RNA sample from array B had a higher concentration of bee brain and thorax than the first. Click here for file

Additional data file 7 There is a different worksheet containing the results of 10 replicates each, for honey bee and *Drosophila*. Each worksheet provides E-score, Bonferroni corrected E-score, and GO term. Click here for file

Additional data file 8 Folded hairpins for precursors of novel honey bee miRNAs. Click here for file

Additional data file 9 Figure A shows a 15% PAGE separation of small-enriched RNAs from honey bee queen head, thorax, and abdomen, and worker head, thorax, and abdomen. RNA sized at 18-30 nt was excised from the gel and purified for qPCR as described in the text. The left lane shows a 10 nt RNA size marker, with the 10 nt band at bottom left. Figure B shows the size variation of PCR products generated from small-enriched RNA pools and candidate primers. Most products were approximately 75-90 bp in length. Alphanumeric label refers to sample ID as described in Additional data file 3. Click here for file

Additional data file 10 Values indicate relative expressions levels as log_10 _scale, with SD for three sample replicates, as described in the text. Click here for file
