# Supplementary material for: Computational and transcriptional evidence for microRNAs in the honey bee genome
Source: Genome Biol. 2007 Jun 1;8(6):R97. doi: 10.1186/gb-2007-8-6-r97 (PMC2394756; doi:10.1186/gb-2007-8-6-r97)
Supplement: Additional data file 8 — Folded hairpins for precursors of novel honey bee miRNAs. [file gb-2007-8-6-r97-S8.pdf]

```
C5152a
dG = -18.30

              10             20             30                40
GAUUUU--     ACCA   A    ua              --| c      CGAUUCUU
          UCUG      CC uaa  auauguuugau   uc uggU           \
          AGAC      GG AUU  UAUACAAACUA   AG ACCA           U
UGUUCAGC     CAAA   C    C-              UA^ A      ACAAAAAA
.            90             80              70             60            50
```

```
C5152b
dG = -37.50

          10             20               30             40             50
CAAACAAGUCG      GU U         -|              au            u     UU
               UCUG   U CCGUAa gauauguuugau   ucuugguug uUU    \
               AGAC   G GGUAUU UUAUACAAACUA   AGGACCAGC AAG    A
AAA--------      UG U         A^              --            U     AA
.                      90             80              70             60
```

```
C5560
dG = -45.90

                          10             20             30               40
A-----------        CU           Aa            g              -| UGCAU
                CUUAA   GGGGUCA   uugacucua uagggaguc cC        U
                GAAUU   CCUCAGU   GACUGAGAU AUCCUUCAG GG        C
AGGCAACUUGCA        UU           CG            A              C^ UAUAA
.              90             80             70             60             50
```

```
C5599
dG = -23.80

          10               20             30
CA      AC     AU---      GGU     A-           .-UG|     g
   CCUG    CUG       CCU     UCG     UUUUUGU       caggu a
   GGAC    GAC       GGA     AGU     AAGAACA       gucua a
G-      --     GCGGC      AGU     AG           \  --^     a
.                 90             80             70         40


                                                     50
                                             g----      agaA
                                                  uucc      \
                                                  GAGG      U
                                             UAAAU      AAGA
                                                        60
```

```
C689
dG = -46.80

           10             20             30             40             50
CAU|     UU      U       a        ua       a         AUGAUUG       AA
    CGCG    GCC   CUuca   uuccg    gugc   uugcag            UUCG     U
    GCGC    CGG   GGAGU   AGGGC    CACG   AACGUC            GAGC     U
CU-^     UC      U       G        GC       -         CAAGAAA       AG
.               90             80               70             60
```

```
C3345
Initial dG =     -32.20

           10              20             30             40
AGAUAAAAGC-     -|                    a      cu          a      AUGU
                GUG GUAUUUGuuuuaga uuc   acgcuuu ccG        U
                UAC CGUAAGCAAAGUCU AAG   UGCGAAA GGU        C
CUACUUCCAAA     G^                    A      UU          C      GAAG
.               90             80             70             60             50
```

```
C1504
dG = -30.10

               10              20
U                    .-A|         au
 CUGUCCUU--GC      UGCaggg     u
 GACGGGAG   CG      auguuuu    c
-             \     \ -^         gg
 .                         30


                                    40
                           acauuc      AA
                                     gcA   \
                                     CGU   U
                           CAGU--      GA
                             50


                       60             70
                  GGAC-     -  A     AGA
                         CGG  GC CGUG    G
                         GCC  CG GCAC    G
                  UUGGU     A  -     AUC
                   90             80
```
