# Supplementary figures and images for: Computational and transcriptional evidence for microRNAs in the honey bee genome
Source: Genome Biol. 2007 Jun 1;8(6):R97. doi: 10.1186/gb-2007-8-6-r97 (PMC2394756; doi:10.1186/gb-2007-8-6-r97)

A)

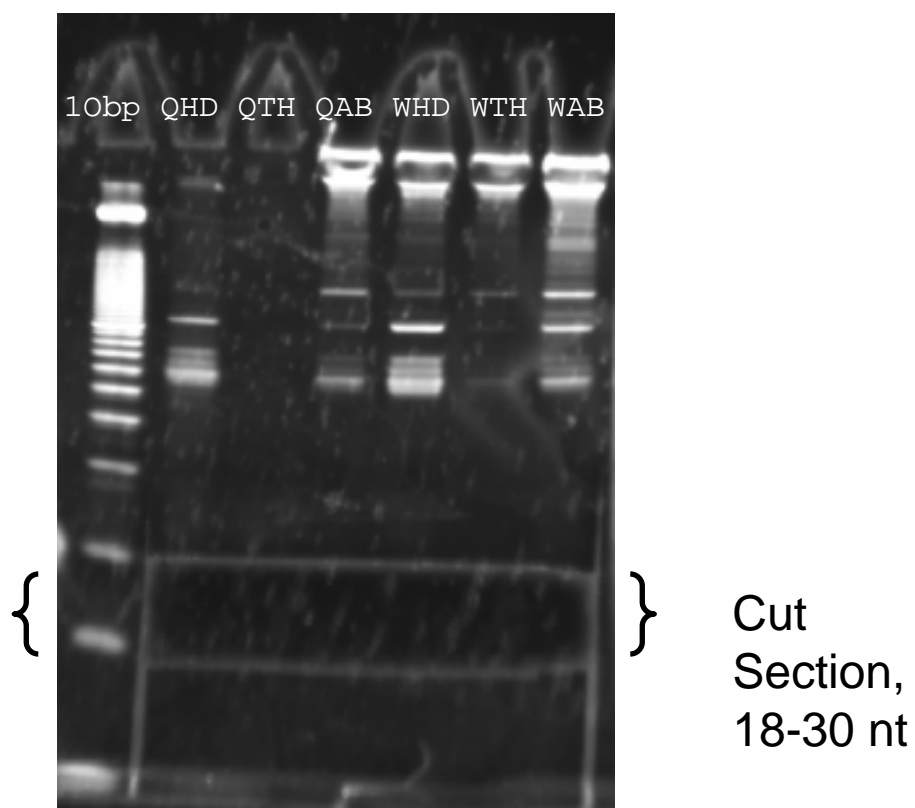

B)

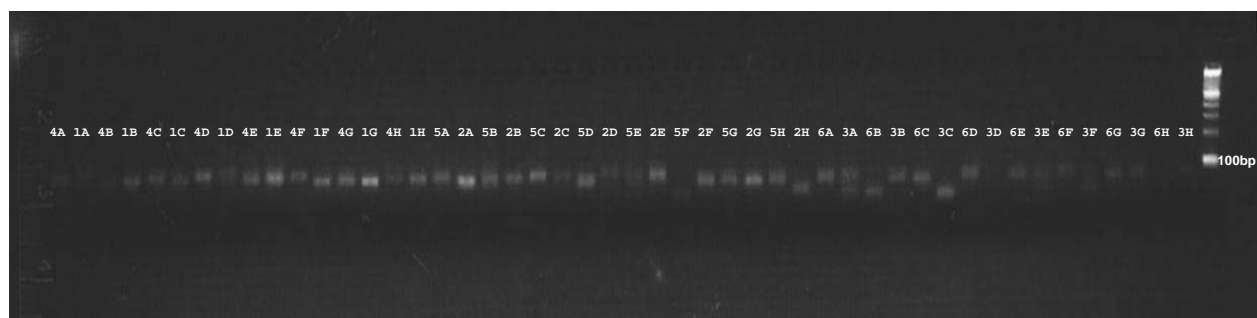

Supplement: Additional data file 9 — Figure A shows a 15% PAGE separation of small-enriched RNAs from honey bee queen head, thorax, and abdomen, and worker head, thorax, and abdomen. RNA sized at 18-30 nt was excised from the gel and purified for qPCR as described in the text. The left lane shows a 10 nt RNA size marker, with the 10 nt band at bottom left. Figure B shows the size variation of PCR products generated from small-enriched RNA pools and candidate primers. Most products were approximately 75-90 bp in length. Alphanumeric label refers to sample ID as described in Additional data file 3. [file gb-2007-8-6-r97-S9.pdf]

Fig. S3

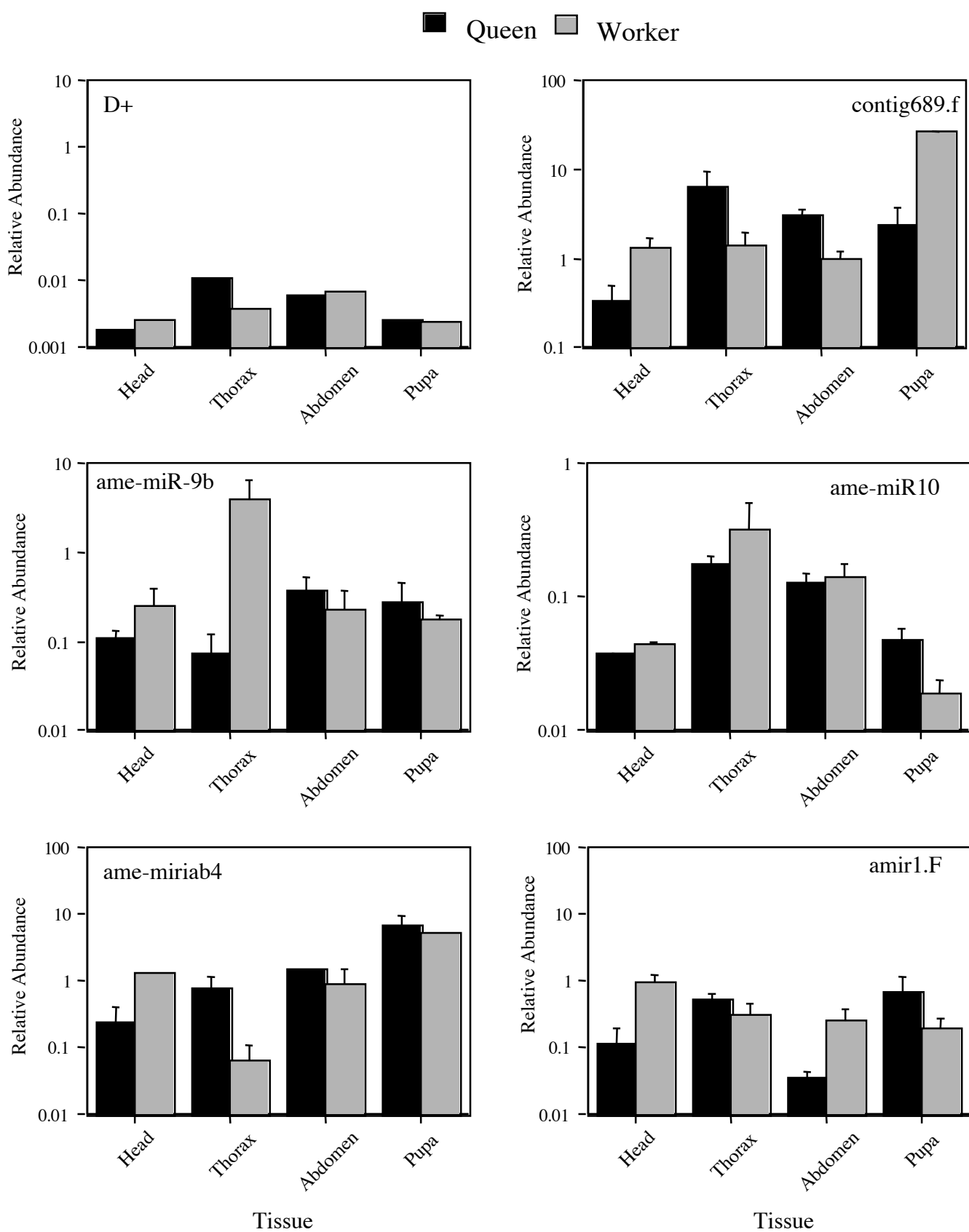

Fig. S1b

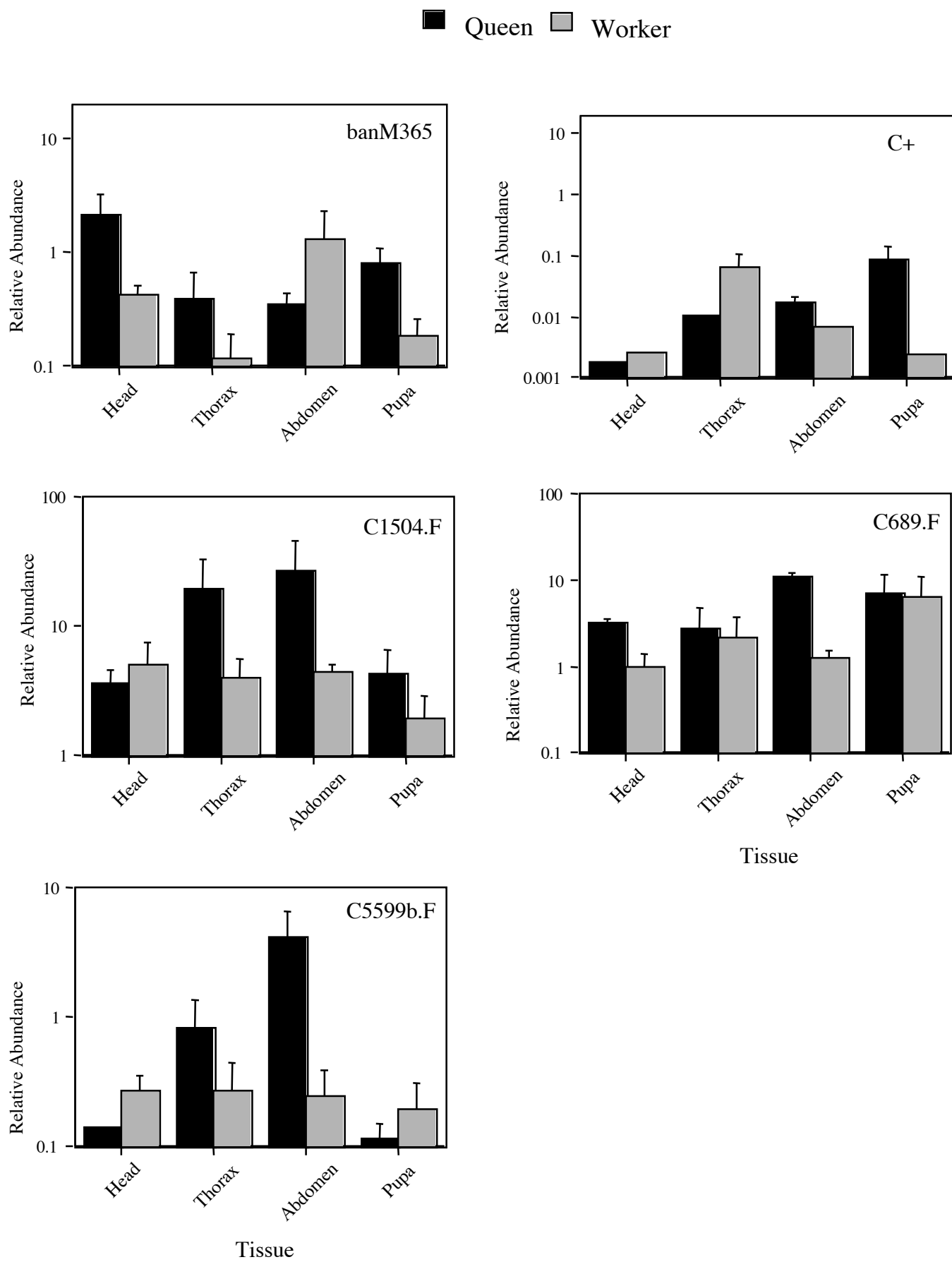

Supplement: Additional data file 10 — Values indicate relative expressions levels as log10 scale, with SD for three sample replicates, as described in the text. [file gb-2007-8-6-r97-S10.pdf]
